# Transcriptome-wide *N*^*6*^-methyladenosine methylome profiling of porcine muscle and adipose tissues reveals a potential mechanism for transcriptional regulation and differential methylation pattern

**DOI:** 10.1186/s12864-017-3719-1

**Published:** 2017-04-28

**Authors:** Xuelian Tao, Jianning Chen, Yanzhi Jiang, Yingying Wei, Yan Chen, Huaming Xu, Li Zhu, Guoqing Tang, Mingzhou Li, Anan Jiang, Surong Shuai, Lin Bai, Haifeng Liu, Jideng Ma, Long Jin, Anxiang Wen, Qin Wang, Guangxiang Zhu, Meng Xie, Jiayun Wu, Tao He, Chunyu Huang, Xiang Gao, Xuewei Li

**Affiliations:** 1 Department of Zoology, College of Life Science, Sichuan Agricultural University, No. 46, Xinkang Road, Ya’an City, 625014 Sichuan Province China; 2 Institute of Animal Genetics and Breeding, College of Animal Science and Technology, Sichuan Agricultural University, Chengdu, Sichuan 611130 China; 3 Genergy Biological Technology (Shanghai) Company of Limited Liability, Shanghai, 200233 China

**Keywords:** Differentially methylated genes, MeRIP-Seq, *N*^6^-methyladenosine, Porcine, Transcriptional regulation

## Abstract

**Background:**

*N* ^*6*^-methyladenosine (m^6^A) is the most prevalent internal form of modification in messenger RNA in higher eukaryotes and potential regulatory functions of reversible m^6^A methylation on mRNA have been revealed by mapping of m^6^A methylomes in several species. m^6^A modification in active gene regulation manifests itself as altered methylation profiles in a tissue-specific manner or in response to changing cellular or species living environment. However, up to date, there has no data on m^6^A porcine transcriptome-wide map and its potential biological roles in adipose deposition and muscle growth.

**Methods:**

In this work, we used methylated RNA immunoprecipitation with next-generation sequencing (MeRIP-Seq) technique to acquire the first ever m^6^A porcine transcriptome-wide map. Transcriptomes of muscle and adipose tissues from three different pig breeds, the wild boar, Landrace, and Rongchang pig, were used to generate these maps.

**Results:**

Our findings show that there were 5,872 and 2,826 m^6^A peaks respectively, in the porcine muscle and adipose tissue transcriptomes. Stop codons, 3′-untranslated regions, and coding regions were found to be mainly enriched for m^6^A peaks. Gene ontology analysis revealed that common m^6^A peaks in nuclear genes are associated with transcriptional factors, suggestive of a relationship between m^6^A mRNA methylation and nuclear genome transcription. Some genes showed tissue- and breed-differential methylation, and have novel biological functions. We also found a relationship between the m^6^A methylation extent and the transcript level, suggesting a regulatory role for m^6^A in gene expression.

**Conclusion:**

This comprehensive map provides a solid basis for the determination of potential functional roles for RNA m^6^A modification in adipose deposition and muscle growth.

**Electronic supplementary material:**

The online version of this article (doi:10.1186/s12864-017-3719-1) contains supplementary material, which is available to authorized users.

## Background

*N* ^*6*^-methyladenosine (m^6^A) is the most prevalent internal form of modification in polyadenylated mRNAs and long-noncoding RNAs in higher eukaryotes, and first identified in the 1970s [1]. It is catalyzed by a multicomponent complex composed of two active methyltransferases, methyltransferase like 3 and methyltransferase like 14. These methyltransferases form a heterodimer that interacts with Wilms′ tumor 1-associating protein and substantially affects mRNA methylation in vivo, but not in vitro [2–6].

Two m^6^A RNA demethylases, fat mass and obesity-associated (FTO) protein [7] and its homolog ALKBH5, which selectively reverse m^6^A to adenosine in nuclear RNA, have been discovered since 2011 [8]. Together with the methyltransferases, they represent the first examples of reversible and dynamic RNA modification similar to DNA and histone methylation, and are an important breakthrough towards reigniting investigations of m^6^A biology [9]. Human YTH domain family 2 was recently identified as the first m^6^A reader protein that preferentially and specifically recognizes m^6^A-methylated mRNA [10, 11] and mediates mRNA decay [11].

Some studies have revealed that RNA m^6^A plays important biological roles in the regulation of cellular metabolic processes. RNA m^6^A controls cell transition fate in mammalian embryonic stem cells [12], regulates pluripotency in murine stem cells [13], and shoot stem cell fate in Arabidopsis [14]. FTO-dependent demethylation of m^6^A regulates mRNA splicing and is required for adipogenesis [15]. m^6^A-methylation-dependent RNA processing controls the speed of the mammalian circadian clock in mice embryonic fibroblasts [16]. m^6^A methylation also plays important roles in human disease, such as control of HIV-1 replication and interaction with the host immune system during HIV-1 infection of T cells [17], promoting translation of oncogenes in human lung cancer [18], and induction of breast cancer stem cell phenotype [19]. Recent studies have proved that m^6^A methylation modulates sex determination in *Drosophila* [20, 21] and promotes X-inactive specific transcript (*XIST*) mediated transcriptional repression in mammal [22].

Understanding the potential biological role of RNA m^6^A modification requires detection of m^6^A modification sites at the transcriptome-wide level. In 2012, Dominissini et al. [10] and Meyer et al. [23] developed a method for transcriptome-wide m^6^A localization, called methylated RNA immunoprecipitation with next-generation sequencing (MeRIP-Seq), and profiled the transcriptome-wide m^6^A distribution in humans and mice. Development of the MeRIP-Seq technique has also enabled transcriptome-wide m^6^A profiling of three other eukaryotic species: *Saccharomyces cerevisiae*, *Arabidopsis thaliana*, and *Oryza sativa* [24–27]. These studies showed that m^6^A is mainly localized around stop codons, 3′-untranslated regions (3′-UTRs), as well as in long internal exons and transcription start sites, suggesting that it plays a key role in the regulation of post-transcriptional gene expression. These groundbreaking studies suggest that it is possible to reveal the potential biological functions of m^6^A modification in other species by constructing transcriptome-wide m^6^A methylome maps.

m^6^A modification in active gene regulation manifests itself as an altered tissue-specific methylation profile. m^6^A modification is widely distributed in animal tissues such as liver, kidney, brain, lung, and heart, and exhibits tissue-specific regulation [10, 23]. For example, in adult mouse brain tissue, genes whose RNAs display m^6^A modifications are linked to neurodevelopmental and neurological disorders [10, 23]. Similarly, transcriptome-wide m^6^A profiling of rice callus and leaf tissues revealed the presence of tissue-specific competitors involved in selective mRNA modification. Selectively methylated genes (SMGs) in callus were mainly found to participate in transcription regulator/factor activity whereas leaf SMGs were mainly involved in plastids and thylakoids [27]. Recently, Wan et al. also found unique differential m^6^A methylation patterns among leaf, flower, and root tissues in *Arabidopsis thaliana* [26].

m^6^A modulation of active gene regulation at the cellular or species level also occurs in response to environmental changes. Dominissini et al. [10] detected a subset of treatment-dependent, dynamically altered peaks in human hepatocellular carcinoma cell line (HepG2) exposed to ultraviolet radiation, heat shock, hepatocyte growth factor, and interferon-c, although these cells exhibited markedly similar m^6^A profiles. Similarly, a comparative study of m^6^A methylation among different geographically diverse accessions of *A. thaliana*, including Can-0 (latitude: 29.21, photosynthetically active radiation (PAR) in spring: 123.7) and Hen-16 (latitude: 65.25, PAR in spring: 55.5), identified strain-specific genes with different biological functions, and also strain-specific m^6^A marking of mRNAs, with higher m^6^A levels in Can-0 than in Hen-16 [25]. These studies indicated that it is possible to reveal the potential biological roles of RNA m^6^A modification by comparative analysis of tissue-specific or population-specific regulation of m^6^A modification.

To further investigate the functions of m^6^A, and to facilitate future studies of mammalian m^6^A, we collected muscle and adipose tissues from three pig breeds with different genetic backgrounds. This enabled us to acquire the first known set of transcriptome-wide m^6^A profiles in pigs. We compared the patterns of m^6^A distribution between muscle and adipose tissues and among porcine breeds, and investigated tissue and breed generality and selectivity of methylated genes and their functional implications.

## Results

### Transcriptome-wide detection of m^6^A modification in pigs

We sampled muscle (LM) and adipose (LA) tissues from two 210-day-old Landrace (LD) sows using the MeRIP-Seq technique. To ensure the specificity of the antibody to m^6^A, we performed dot blot experiments to compare m^6^A-IP (immunoprecipitation) enrichment with control RNA (input, without IP) before sequencing. The results demonstrated that the m^6^A antibody selectively binds to m^6^A residues and exhibits negligible binding to unmodified adenosines (Additional file 1: Figure S1). We obtained more than 37,600,000 reads from each LM IP sample and more than 30,900,000 reads from each LA IP sample. After filtering out low-quality data, more than 37,400,000 high-quality reads from each LM IP sample and more than 30,600,000 high-quality reads from each LA IP sample were mapped to the *Sus scrofa* reference genome. More than 70% of the IP reads from both tissues uniquely mapped to the reference genome. To improve m^6^A peak identification, we also sequenced two input samples for each tissue simultaneously, and acquired ~24,000,000 high-quality reads related to ~16,000 genes from each LM input sample and more than 21,600,000 high-quality reads related to ~17,000 genes from each LA input sample (Additional file 2: Table S1).

**Fig. 1:**
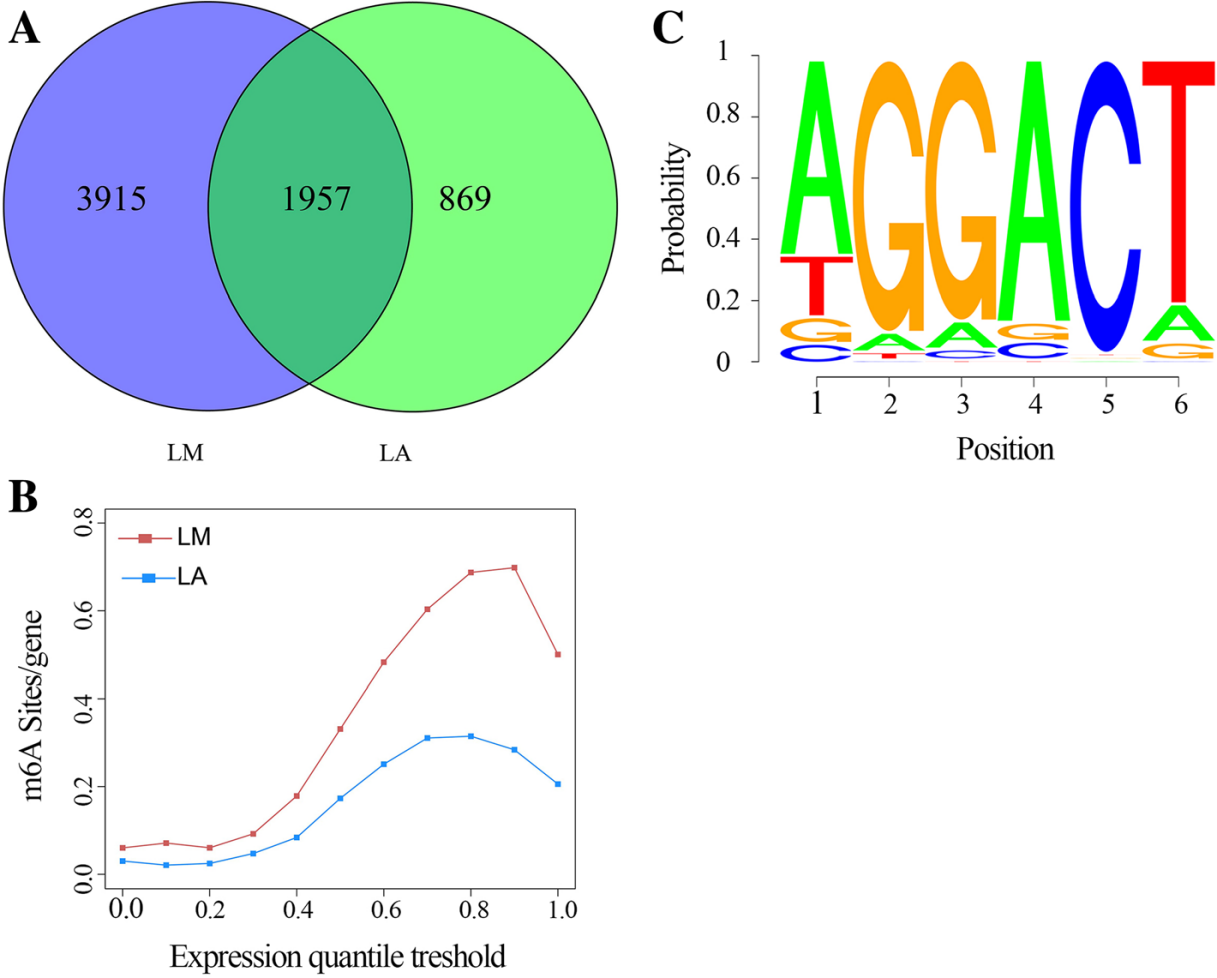
Outline of the porcine m^6^A methylome. **a** Numbers of common and tissue-specific m^6^A peaks in the LM and LA tissues. LM and LA represent muscle and adipose tissues from Landrace pigs respectively (**b**) Estimation of m^6^A peaks density in porcine transcripts. Transcripts were divided to 11 groups based on expression levels, and the m^6^A density of each group was calculated separately. **c** Sequence motif of m^6^A-containing peak regions

For both tissues, more than 80% and 70% of the m^6^A peaks were consistently detected in two biological replicates of LM and LA, respectively. We regarded these recurrent peaks as highly enriched m^6^A peaks for further analysis. We detected 5,872 m^6^A recurrent peaks among 4,544 expressed genes in LM, and 2,826 m^6^A recurrent peaks among 2,137 expressed genes in LA (Table 1 and Fig. 1a and Additional file 3: Data S1). We used this information to estimate that the porcine transcriptome contains 0.562 m^6^A peaks in LM and 0.254 m^6^A peaks in LA per actively expressed transcript, respectively (Fig. 1b and Additional file 2: Table S2).

**Table 1:** Summary of m^6^A peaks in muscle and adipose tissues and their distribution

Sample	Total identified peaks	Coding genes	% peaks in 5'UTR segment	% peaks in start codon segment	% peaks in CDS segment	% peaks in stop codon segment	% peaks in 3'UTR segment	% peaks in others segment
LM1	7131	5864	14.61	16.43	27.85	34.51	24.42	17.45
LM2	6603	5364	14.23	16.36	25.56	36.34	25.68	18.28
LM	5872	4544	14.46	16.93	23.23	38.55	17.82	20.43
LA1	3982	3303	8.56	10.41	36.81	30.88	22.07	16.29
LA2	3575	2974	8.58	10.68	35.09	30.47	23.73	16.83
LA	2826	2137	8.75	11.14	30.82	34.07	13.25	23.64

LM1 and LM2 mean the sample 1 and sample 2 of muscle tissue from Landrace pigs, respectively; LM is the recurrent peak sample for LM1 and LM2 (≥50% overlapping lengths); LA1 and LA2 mean the sample 1 and sample 2 of adipose tissue from Landrace pigs, respectively; LA is the recurrent peak sample for LA1 and LA2 (≥50% overlapping lengths); UTR, untranslated region; CDS, coding sequence

To determine whether our identified m^6^A peaks shared the conserved RRACH motif (where R represents purine, A is m^6^A and H is a non-guanine bas) [28, 29], we performed an unbiased search for motifs enriched in regions surrounding m^6^A peaks (Methods). Clustering of significantly enriched sequences perfectly recapitulated the previously established m^6^A RRACH consensus sequence in both tissues (Fig. 1c and Additional file 1: Figure S2). The identification of a strong consensus reinforces the authenticity of the discovered m^6^A peaks, and supports the existence of a predominant methylation machinery.

### Distribution of m^6^A modification in the porcine transcriptome

**Fig. 2:**
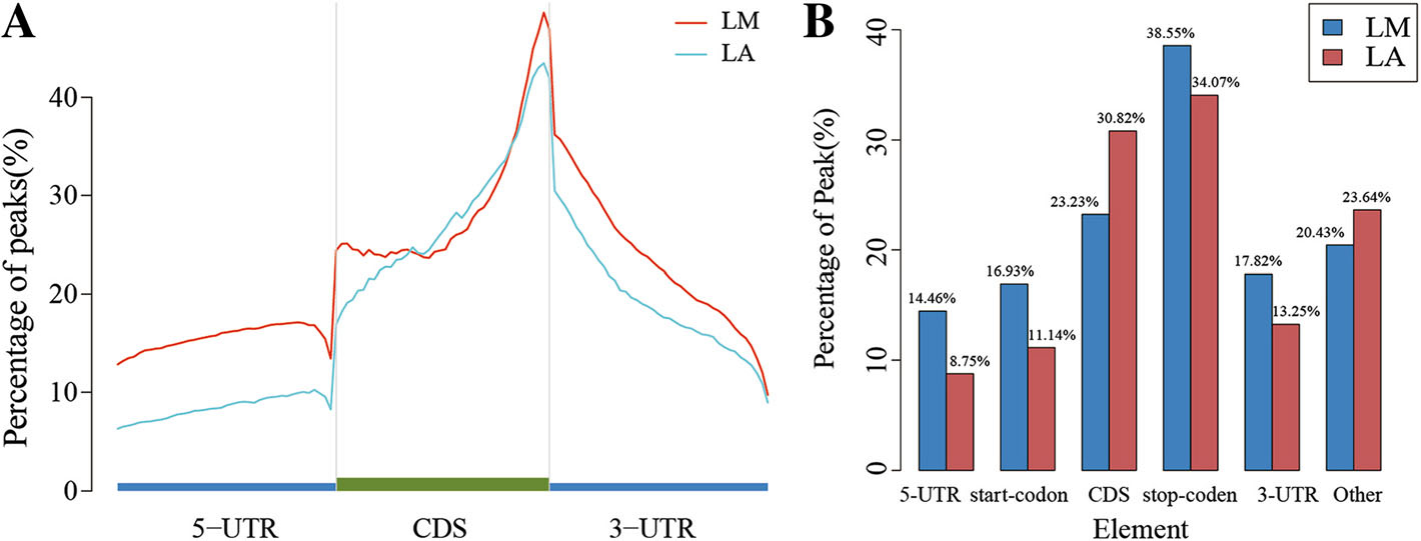
Distribution of m^6^A methylome along porcine transcripts. **a** Enrichment of m^6^A peaks along transcripts. Each transcript is divided into three parts: 5′UTR, CDS and 3′UTR. **b** Transcriptome-wide distribution of m^6^A peaks. Bar graph denotes the percentage of m^6^A peaks in each of the six non-overlapping transcript elements

To understand the preferential location of m^6^A in transcripts, we next investigated the metagene profiles of m^6^A peaks in the entire transcriptome of both tissues. We observed that m^6^A peaks were markedly correlated with two distinct coordinates: immediately following near the end of the 5′untranslated regions (5′UTRs) and start of the coding sequence (CDS), and near the end of the CDS and beginning of 3′untranslated region (3′UTRs) in both tissues (Fig. 2a); meanwhile, the end CDS of peaks were more pronounced than the start CDS of peaks. To assess the enrichment methodically, we assigned each m^6^A peak to one of six no overlapping transcript segments: 5′UTRs, start codon, CDS, stop codon, 3′UTR, and other (Fig. 2b). Most (~80%) of the m^6^A peaks were within genic regions, and more than 60% of genic peaks were localized near the stop codon and CDS, while ~ 30% were found in the 5′UTRs, start codons, and 3′UTRs (Fig. 2b). The topological patterns distributing within genes were highly similar in both tissues, suggesting that recognition of motif for m^6^A methylation was conserved among animal tissues.

### m^6^A-containing genes are associated with transcriptional factors and involved in important biological pathways

**Fig. 3:**
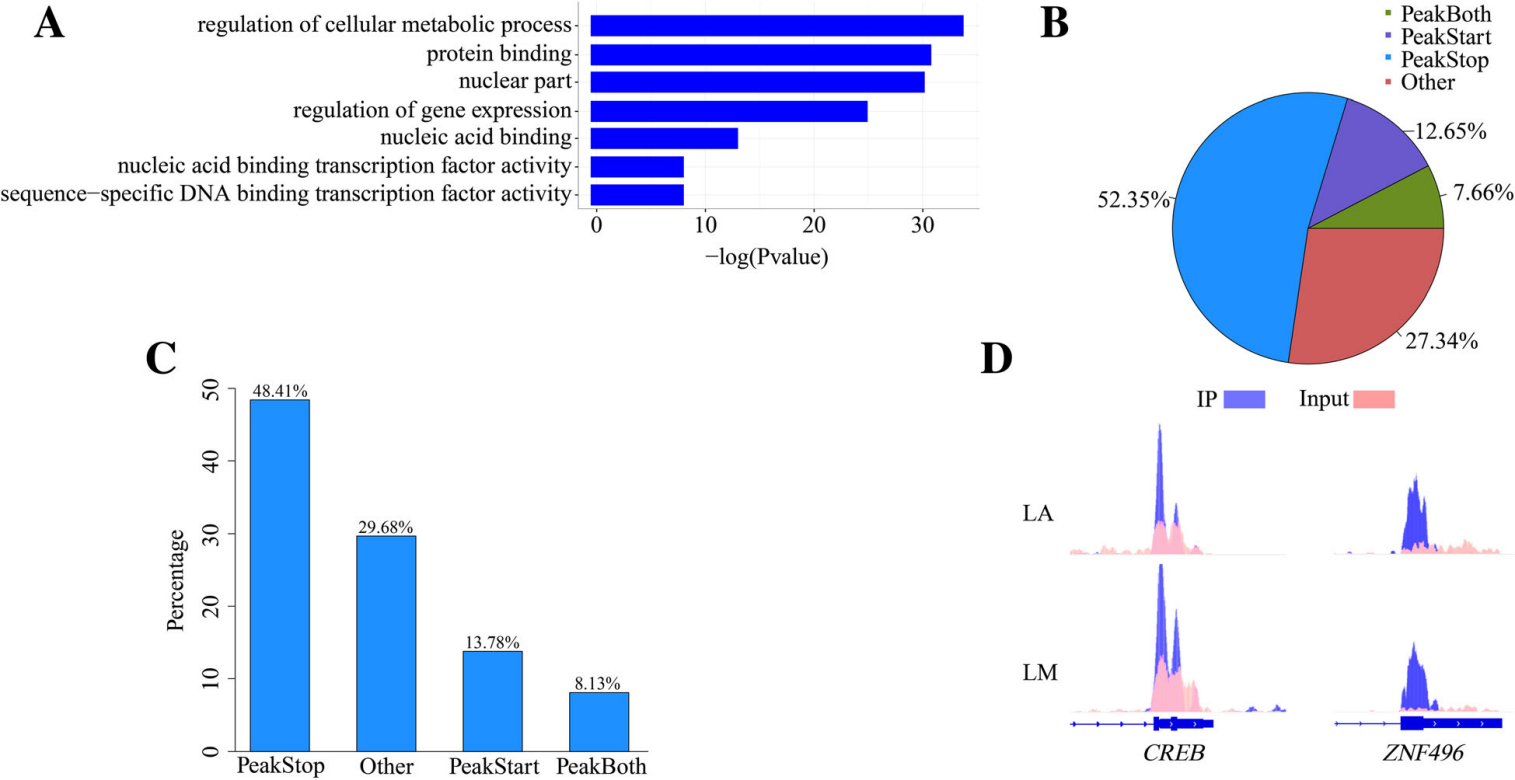
Functional analysis of common genes associated with m^6^A peaks. **a** Gene ontology analysis of common genes associated with m^6^A peaks. Gene ontology (GO) categories are maintained by the Gene Ontology Consortium and *P* values are calculated using the DAVID tool. **b** Percentages of subgroups of genes divided by the localization pattern of m^6^A peaks. **c** Percentages of nucleus-related genes in each subgroup. **d** Examples of nucleus-related genes with m^6^A peaks around the stop codon

To further determine general functional pathways that involve m^6^A in animal tissue development, we systematically screened these common peaks which consistently appears on both tissues (≥50% overlapping lengths) and these related common expression genes, and identified the GO terms with the help of the GO consortium database (Methods). We identified 1,957 common m^6^A peaks representing 1,615 expressed genes in the two tissues (Fig. 1a and Additional file 4: Data S2). GO analysis showed that the genes encoding m^6^A-containing RNAs are mainly enriched in the nucleus, and are involved in a variety of cellular functions including protein binding, nucleic acid binding, transcription factor activity, sequence-specific DNA binding, transcription factor activity and regulation of cellular metabolic process (Fig. 3a and Additional file 4: Data S2).

With regards to genes associated with m^6^A peaks, we sought to determine if differential m^6^A enrichment regions are related to specific GO categories. We classified genes into four subgroups: PeakStart (m^6^A peaks around the start codon), PeakStop (m^6^A peaks around the stop codon), PeakBoth (m^6^A peaks around both start and stop codons) and others (Fig. 3b). We then performed GO enrichment analysis for each subgroup. All subgroups of m^6^A-containing genes were found to be highly enriched for cellular components related to the nucleus, while more than 48% of genes belonging to the PeakStop subgroup were enriched for nuclear components (Fig. 3c).

These findings prompted us to analyze the nucleus-related genes in the PeakStop subgroup. We found that most of these genes encode transcription factors (Additional file 5: Data S3), indicating that m^6^A modification is involved in transcriptional regulation. For instance, our m^6^A-IP data revealed one clear m^6^A peak around the stop codon of the cAMP responsive element-binding protein gene (*CREB*) and ten genes from the zinc finger protein (ZNF), including *ZNF496* (Fig. 3d). *CREB* was first described as a cAMP-responsive transcription factor regulating the somatostatin gene in 1987 [30], while ZNF is regarded as one of the most important eukaryotic transcription factors [31]. A large fraction of m^6^A-containing genes being associated with transcription factors suggests a relationship between m^6^A mRNA methylation and nuclear genome transcription.

### m^6^A modification is involved in tissue-differential regulation

**Fig. 4:**
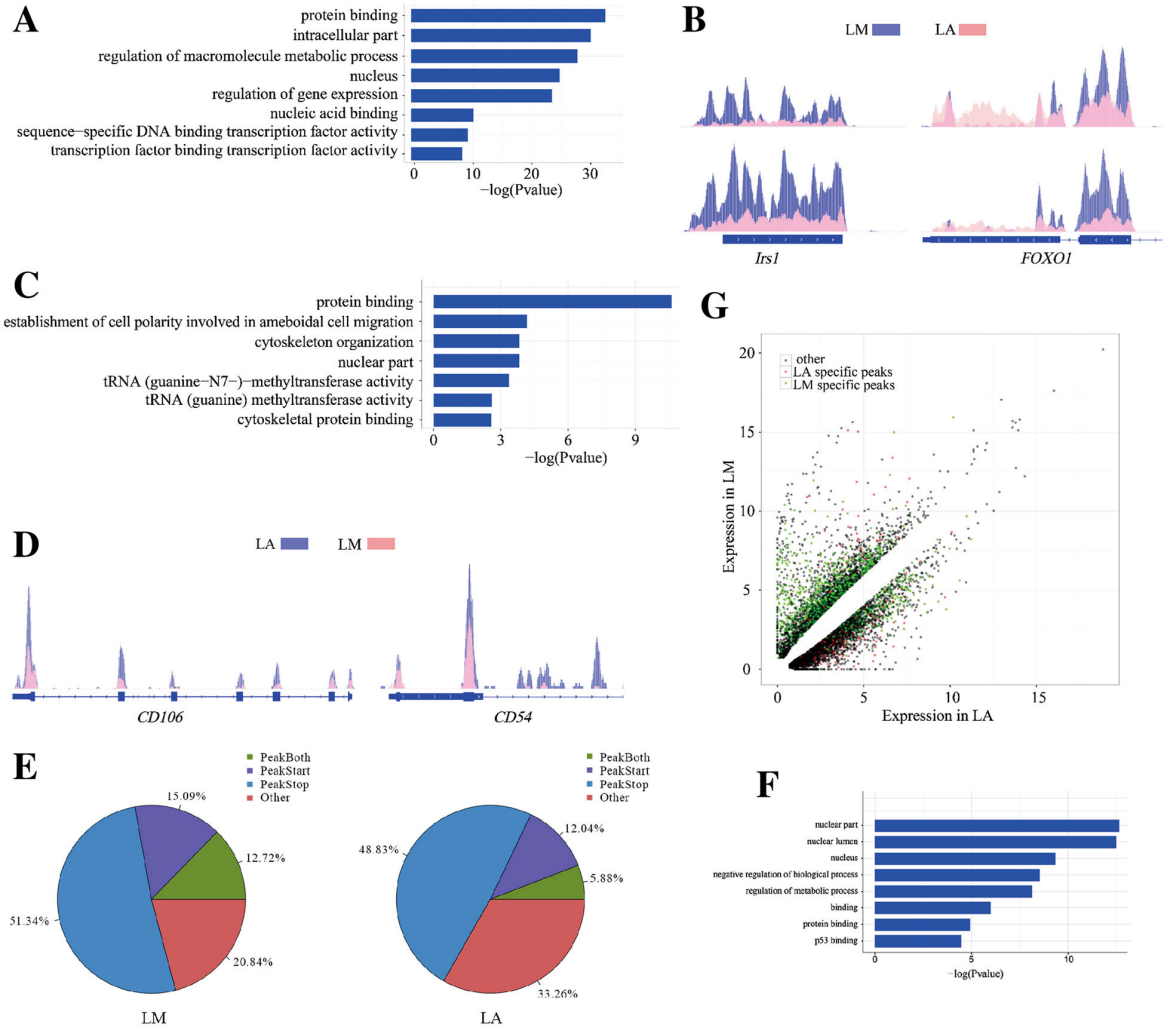
Functional analysis of differentially methylated genes between tissues. **a** Gene ontology analysis of tissue specifically methylated genes. **b** Examples of tissue specifically methylated genes with m^6^A peaks. **c** Gene ontology analysis of tissue dynamic methylated genes. **d** Examples of tissue dynamic methylated genes with m^6^A peaks. **e** Percentages of subgroups of genes divided by the differential peaks in gene regions between the two tissues. **f** Gene ontology analysis of differential peaks in gene regions related methylated genes between the two tissues. **g** Differentially expressed mRNAs in LM and LA. Genes with LM specific m^6^A peaks are highlighted in green, genes with LA specific m^6^A peaks are highlighted in orange, and genes with other m^6^A peaks are highlighted in black

Although some m^6^A peaks are shared between both tissues, we could detect a proportion of tissue-differential peaks (Methods). We firstly screened tissue-specific peaks between LM and LA, and identified 3,915 LM-specific and 869 LA-specific peaks (Fig. 1a). These peaks represented 3,034 and 562 tissue specifically methylated genes (TSMGs) in LM and LA respectively (Additional file 6: Data S4). GO analysis showed that these TSMGs are mainly involved in intracellular protein or nucleic acid binding, regulation of macromolecule metabolic processes and gene expression (Fig. 4a and Additional file 7: Data S5). LM TSMGs are mainly involved in the regulation of energy dependent signaling pathways such as the insulin signaling pathway and the AMP-activated protein kinase signaling pathway, while LA TSMGs are mainly involved in fatty acid metabolism such as the glyoxylate and lipoic acid metabolism, as well as cytoskeleton and system development. For example, our m^6^A-IP data revealed significant m^6^A peaks around the 5′UTR, stop codon and CDS of *Irs1* and *Foxo1* mRNAs from the insulin signaling pathway in LM but not in LA (Fig. 4b). The Irs1/2 → PI3K → Akt → Foxo1 branch of the insulin signaling pathway is largely responsible for hepatic insulin-regulated glucose homeostasis and somatic growth [32].

Further, we analyzed the dynamic m^6^A peaks, which were common to both tissue types but showed altered intensities judged by MAnorm model (Methods). We identified 382 common m^6^A peaks of significantly differential intensities, which represented 319 tissue dynamic methylated genes (TDMGs) in the two tissues (Additional file 6: Data S4). GO analysis showed that most TDMGs are involved in protein binding and establishment of cell polarity responsible for amoeboid cell migration, while some are involved in the regulation of immune and disease-related signaling pathways (Fig. 4c and Additional file 7: Data S5). Our m^6^A-IP data revealed significant m^6^A peaks in LA, but not LM, for five genes from the immunoglobulin superfamily (IGSF) including *CD54* (*ICAM-1*), and *CD106* (*VCAM1*) (Fig. 4d), which promote the adhesion of inflammatory sites, and play an important role in tumor progression and metastasis, and regulates immune responses [33].

Besides, we searched for differential peaks in gene regions between the two tissues. We found that LM had more peaks than LA, which were located in stop codon/ start codon/ both regions (Fig. 4e and Additional file 6: Data S4). GO analysis revealed that these tissue-differential methylated genes are mainly enriched for the nuclear part and are involved in the regulation of metabolic processes and protein binding (Fig. 4f and Additional file 7: Data S5).

As an initial exploration into the functional implications of m^6^A methylation differences across genomes, we asked whether m^6^A methylation regulates differences in gene expression. Using the input RNA-Seq data, we investigated the differential expression of genes (DE genes) from the two tissues. We identified 2,988 genes that were highly expressed in LM and 3,264 that were highly expressed in LA. Within the LM-high list, we detected more genes that contained m^6^A peaks in LM than in LA (1012/312, *P* < 0.001, Fisher’s exact test). However, in the list of LA-high genes, we detected less genes containing m^6^A peaks in LA than in LM (495/581, *P* < 0.001, Fisher’s exact test) (Fig. 4g and Additional file 2: Table S3 and Additional file 6: Data S4). These results indicated that each tissue possesses its own characteristic m^6^A methylation sites that appear to be associated with gene activation.

### m^6^A modification occurs in a breed-differential pattern

m^6^A modulation of active gene regulation at the cellular or species level also occurs in response to environmental changes and genetic background differences [10, 25], these findings indicated that it is possible to reveal the potential biological roles of RNA m^6^A modification by comparative analysis of pig breed-differential regulation of m^6^A modification. To explore the patterns and functions of m^6^A methylation modification among different pig breeds, we sampled muscle tissues from three pig breeds with different genetic backgrounds using the MeRIP-Seq technique. The three breeds included the undomesticated wild boar (WB, *S.s. moupinensis*), and the domestic Landrace (LD; a leaner, Western breed) and Rongchang (RC; a fatty, Chinese breed) pig breeds. We detected approximately 6, 500 ~ 7,500 m^6^A peaks representing approximately 5,500 ~ 6,000 expressed genes among the three breeds (Additional file 8: Data S6). The data revealed that WB showed a highest total m^6^A level while a lowest total m^6^A level was found in RC.

To discover the differential m^6^A methylation across the three pig breeds, we firstly identified multiple breed-specifically methylated peaks and breed-specifically methylated genes (BSMGs). Between WB and LD, 2,155 and 1,843 specific peaks were found, representing 2,846 (1,452/1,394) BSMGs, respectively (Additional file 1: Figures S3A and Additional file 9: Data S7). Likewise, between WB and RC, 2,510 and 1,912 specific peaks were found, representing 3186 (1,733/1,453) BSMGs, respectively (Additional file 1: Figures S3B and Additional file 9: Data S7); while 1,709 and 1,432 specific peaks were obtained between RC and LD, representing 2285 (1,257/1,028) BSMGs, respectively (Additional file 1: Figures S3C and Additional file 9: Data S7). GO analysis indicated that these BSMGs are mainly enriched for intracellular processes and are involved in fundamental biological functions such as protein/nucleic acid binding, transcription factor binding, cellular metabolic processes, developmental processes, and positive regulation of biological processes (Additional file 1: Figures S4A and S4B and S4C and Additional file 10: Data S8).

**Fig. 5:**
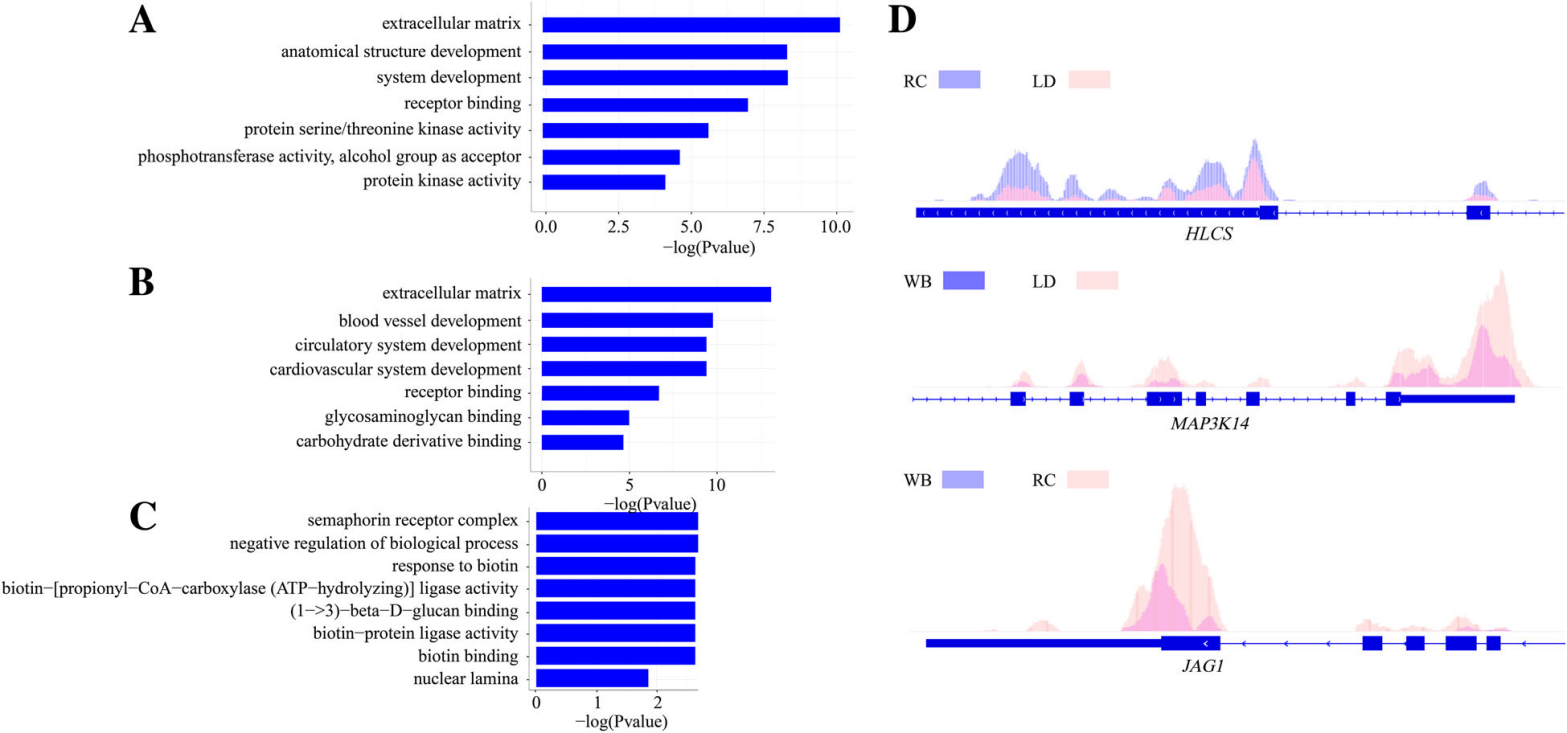
Functional analysis of differentially methylated genes among breeds. **a** Gene ontology analysis of breed dynamic methylated genes between WD and LD. WD represents Wild boar and LD represents Landrace. **b** Gene ontology analysis of breed dynamic methylated genes between WD and RC. RC represents Rongchang pigs. **c** Gene ontology analysis of breed dynamic methylated genes between RC and LD. **d** Examples of breed dynamic methylated genes with m^6^A peaks

We further searched for the dynamic m^6^A peaks, which were common between both breeds but showed altered intensities judged by MAnorm model (Methods), and identified the dynamic m^6^A peaks related genes defined as breed dynamic methylated genes (BDMGs). First, between WB and LD, we found 219 common m^6^A peaks of significantly different intensities, representing 171 BDMGs (Additional file 9: Data S7). GO analysis indicated that these BDMGs are mainly enriched for the extracellular matrix and are involved in protein kinase, protein serine/threonine kinase and phosphotransferase activities (Figs. 5a and Additional file 10: Data S8). For instance, our m^6^A-IP data revealed significant m^6^A peaks around the 3′UTRs of *MAP3K14* (mitogen-activated protein kinase 14) in LD but not in WB (Fig. 5d). MAP3K14 is a serine/threonine protein-kinase that stimulates nuclear factor κB activity [34]. Second, we found 296 common m^6^A peaks of significantly different intensities between WB and RC, representing 240 BDMGs (Additional file 9: Data S7). GO analysis indicated that these BDMGs are mainly enriched for the extracellular matrix and are involved in receptor/protein/glycosaminoglycan/binding, and development of the cardiovascular system (Fig. 5b and Additional file 10: Data S8). Our m^6^A-IP data revealed significant m^6^A peaks around the stop codon of *JAG1* (cardiac transcription factor Jagged1) in RC but not in WB (Fig. 5d). JAG1 plays an important role in cardiac development [35]. Similarly, we obtained 112 common m^6^A peaks of significantly different intensities, representing 89 BDMGs between RC and LD (Additional file 9: Data S7). GO analysis indicated that these BDMGs are mainly enriched in biotin binding/biotin-protein ligase activity, semaphorin receptor complex/nuclear lamina, biotin-related metabolic pathway, and negative regulation of biological response to biotin (Fig. 5c and Additional file 10: Data S8). Our m^6^A-IP data revealed significant m^6^A peaks around the 5′UTRs of *HLCS* (holocarboxylase synthetase) in RC but not in LD (Fig. 5d). HLCS is the only enzyme in the human proteome capable of catalyzing the binding of biotin to proteins [36].

## Discussion

### m^6^A topological patterns between mammal and plant

In this study, we produced the first porcine transcriptome-wide m^6^A modification profile using MeRIP-Seq technology, and discovered that pig mRNA m^6^A sites are mainly enriched around stop codons, CDS, and 3′UTRs. They share a distribution similar to those of humans and mice by being enriched in long exons, near stop codons, and in 3′UTRs, although they also occur in 5′UTRs [10, 23]. This indicates that the overall distribution of m^6^A sites is similar in the mammalian transcriptomes. m^6^A sites in plants are not only enriched around stop codons and within 3′UTRs but are also found around start codons and 5′UTRs [25, 27]. This difference in distribution is suggestive of species-specificity of this form of mRNA methylation, nevertheless the difference in m^6^A enrichment pattern needs further confirmation. Overall, dominant m^6^A enrichment near stop codons and 3′UTRs is shown in most of mRNA between mammal and plant in this study as previously reported [10, 12, 23, 25–27], and this m^6^A distributing type may represent the typical m^6^A topological pattern in most of the mature mRNA. The extensively higher m^6^A signals at the stop codon or 3′UTRs may be responsible for RNA stability, signaling for transport and translocation, or as regulatory elements for protein translation through the recruitment of specific factors onto these m^6^A sites for RNA transport or protein synthesis [11, 37].

The consensus motif sequence RRACH has previously been shown to be over-represented in m^6^A motif regions [38–40] and also further been identified in some high throughput m^6^A RNA sequencing databases [10, 23, 25, 26]. Accordingly, in our current study we successfully identified the consensus motif sequence in the porcine transcriptome. Interestingly, Li et al. failed to find this consensus sequence RRACH in m^6^A motif regions of rice but another different motif sequences were enriched both by MEME and HOMER software [27]. It is uncertain of if the consensus sequence of the methylation in plants are different from mammals, so more methylome data of plant are required to confirm this hypothesis.

### Extensively high m^6^A methylation in certain transcripts may be suitable or required for the transcriptional regulations of these transcripts

Several studies have reported that RNA m^6^A methylation plays a key role in the regulation of post-transcriptional gene expression [10, 23, 41, 42] and the importance of m^6^A in post-transcriptional regulation of gene expression is further reinforced by the discovery and characterization of mammalian reader proteins that recognize m^6^A modifications of mRNA and subsequently affect the stability of the target transcripts [11] In this study, extensive high m^6^A methylation was also found in the transcripts for hundreds of nuclear signaling factors and transcription factors, such as CREB described as a cAMP-responsive transcription factor regulating the somatostatin gene [30] and ZNF family members regarded as one of the most important eukaryotic transcription factors [31], suggesting a relationship between m^6^A mRNA methylation and nuclear genome transcription. High methylation in these transcripts for nuclear transcription factors may confer signaling recognition to these transcripts [10].

Earlier findings revealed that one of the main functions of m^6^A is to mediate mRNA degradation in mammalian cells [4–6, 11], suggesting a possible negative relationship between the m^6^A methylation extent and the transcript level. Consequently, we also discovered that the highly expressed transcripts from adipose tissue were less methylated by m^6^A, as previously found in human brain and mouse liver tissues [10]. However, this observation somewhat differed from our present result in muscle tissue and these reports in leaves, flowers, and roots of *A. thaliana*, which showed that most of the highly expressed transcripts were relatively more modified by m^6^A [25, 26]. The reasons causing the differences may be due to different methodologies, different biological species, or different tissue samples although a number of studies have proved that m^6^A methylation is highly conserved among eukaryotes [10, 23, 25–27, 30]. Our results indicated that each tissue may possess its own characteristic m^6^A methylation sites, suggesting a regulatory role for m^6^A in gene expression.

### Potential roles of differential m^6^A methylation among cells, tissues, and organs

m^6^A-modified nucleotides were previously shown to be widely distributed in animal tissues including the liver, kidney, brain, lung, and heart [10, 23]. Here, we detected 0.562 and 0.254 m^6^A peaks per actively expressed transcript in pig muscle and adipose tissues respectively. These results further affirm our notion that m^6^A is a universal form of RNA modification in animal tissues. However, the differential levels of m^6^A methylaytion in both tissues were much lesser than previous estimations of approximately 2 ~ 3 m^6^A residues per average mRNA transcript in mammalian cells, such as human hepatocellular carcinoma cell line (HepG2) [10], mouse embryonic rat brain tissue [23], mouse naïve embryonic stem cells (ESCs), embryoid bodies (EBs) and embryonic fibroblasts (MEFs) [43], and mouse 3 T3-L1 pre-adipocyte cell line [15]. The differential proportion of the m^6^A modified transcripts may be due to different cell or tissue type, suggesting that these cells and tissues with strong proliferation and differentiation may require higher levels of m^6^A methylaytion to adapt to faster growth and development.

Differential m^6^A methylaytion among tissues has proved responsible for tissue or organ differentiation and development. For example, in adult mouse brain tissue, genes encoding m^6^A-containing RNAs are linked to neurodevelopmental and neurological disorders [10, 23] whereas m^6^A RNA methylation in *Drosophila* and Zebrafish early embryogenesis shows a conserved mechanism of neuronal mRNA regulation contributing to brain function [21, 44]. In plants, callus selectively methylated genes (SMGs) mainly participate in transcription regulator/factor activity whereas leaf SMGs mainly involve plastids and thylakoids [27]. Similarly, differential m^6^A patterns across organs including leaf, flower, and root organs were also found in *Arabidopsis* [26]. Here, we uncover tissue-differential regulation roles of m^6^A modification in muscle and adipose tissues. Differentially methylated genes of LM mostly participate in the regulation of energy-dependent signaling pathways, while those of LA regulate immune- and disease-related signaling pathways. This is similar to the previous observation that differentially DNA methylated regions are associated with obesity or cardiovascular diseases in porcine adipose tissue [45].

### Potential roles of differential m^6^A methylation among populations

Modifications to transfer RNA and ribosomal RNA have previously been shown to change in response to stimuli, suggesting a general model of dynamic control for RNA modification [46, 47]. Similarly, m^6^A RNA modification also exhibits population-specific regulation in response to changes in cellular or species environment. Dominissini et al. [10] observed a subset of treatment-dependent, dynamically altered peaks in different human HepG2 cell lines. Similarly, Luo et al. [25] found that geographically diverse accessions of *A. thaliana* show strain-specific m^6^A mRNA imprints, with the strain-specific genes possessing different biological functions. Li et al. [45] detected differentially methylated DNA regions between LD, Tibetan, and RC pig breeds. In the present study, we also observed breed-specific regulation of m^6^A RNA modification. There are differences among the three porcine breeds with a highest total m^6^A level in WB and a lowest total m^6^A level in RC, and the m^6^A distribution could be influenced by differences in the breed’s surrounding environment and genetic backgrounds. The surrounding living environment for undomesticated WB is more complex than that for domestic LD and RC breeds, which may require a higher proportion of the m^6^A transcripts in wild boar to adapt to more diverse conditions; however, the genetic diversity of RC breed is lower because of long term artificial selection and small population number, which may require a lower extent of m^6^A RNA methylation in the Rongchang pig breeds to fit for these metabolisms that were processed in severe artificial selection conditions and small population in most of cases if our hypothesis aforementioned is rational.

Differential m^6^A methylaytion among breeds showed a connection of the functions of these transcripts required for or specific to this breed. For example, the transcripts presenting an extensively higher level of m^6^A methylation in LD were related to protein kinase, protein serine/threonine kinase and phosphotransferase activities, which may contribute to growth and development of muscle and to adapt to lean LD breeding. However, some of the differential and extensively methylated transcripts in the fatty RC pig were involved in the development of the cardiovascular system, further suggesting that there is a strong relationship between obesity and cardiovascular disease [45]. Briefly, the different m^6^A modification patterns may reflect marked phenotypic changes between WB, the leaner LD, and the fatty RC pig breeds. Outbreeding of these lineages is known to result in differences at the genetic level, the epigenetic state, as well as potential genotype–epigenotype interactions [48].

## Conclusions

We provide the first known porcine transcriptome-wide m^6^A map of differentiated tissues and breeds. Our map reveals features of m^6^A distribution in the porcine transcriptome, and identifies tissue and breed generality as well as selectivity of methylated genes and their functional implications. We also discover a relationship between the m^6^A methylation extent and the transcript level, suggesting a regulatory role for m^6^A in gene expression. This comprehensive map provides a solid basis for the determination of potential functional roles for RNA m^6^A modification in adipose deposition and muscle growth.

## Methods

### Animals

Two 210-day-old sows from each of the pig breeds, undomesticated wild boar (WB), domestic Landrace (LD; a leaner, Western breed) and Rongchang (RC; a fatty, Chinese breed), were used in this study, and these pigs derived from the Farm of Sichuan Agricultural University, Ya'an city, Sichuan province, China. The WB originated in Southern China and belongs to *S. s. moupinensis* (a subspecies of wild boar). The WB lived in a wild state and were little artificial selection except for the transient impact of feeding system during the experimental period. There was no direct and collateral blood relationship among the last three generations of the six selected pigs. The piglets were weaned simultaneously at 28 ± 1 days of age. A starter diet administered from the 30^th^ to the 60^th^ day after weaning provided 3.40 Mcal kg^−1^ metabolizable energy with 20.0% crude protein (11.5 g/kg lysine). From the 61^st^ to the 120^th^ day, the pigs were fed a diet containing 14.0 MJ/kg of metabolizable energy consisting of 18.0% crude protein (9.0 g/kg lysine). From the 121^st^ to the 210^th^ day, they received a diet containing 13.5 MJ/kg of metabolizable energy and 16.0% crude protein (8.0 g/kg lysine). The animals were allowed access to food and water *ad libitum* and were maintained under the same conditions.

### Tissue collection

At their predetermined slaughter age, all pigs were transported to the nearby slaughterhouse and humanely sacrificed. These pigs were not fed on the night before slaughter and were allowed to rest for 2 h after about 1 h of transportation (including loading and unloading), after which they were electrically stunned (90 V, 10 s, and 50 Hz) and exsanguinated as necessary to ameliorate suffering. All samples used in this study were collected according to the guidelines for care and use of experimental animals established by the Ministry of Science and Technology of China. Subcutaneous fatty tissue and the *longissimus dorsi* of the left side of each carcass at the last third/fourth rib were rapidly separated and immediately frozen in liquid nitrogen and stored at −80 °C for RNA extraction.

### RNA isolation and fragmentation

Total RNA was extracted from the *longissimus dorsi* and subcutaneous fatty tissue of the three breeds (LD, WB, and RC) using Trizol (Invitrogen, California, USA). Enrichment of polyadenylated RNAs (polyA^+^ RNAs) from total RNA was performed for a single round using the GenElute mRNA miniprep kit (Sigma-Aldrich, St Louis, MO, USA). RNA quality was tested by a nucleic acid concentration detector and gel electrophoresis. Enriched mRNAs were chemically fragmented into ~100-nucleotide fragments by incubating at 94 °C for 5 min in fragmentation buffer (Ambion, Austin, TX, USA). The fragmentation reaction was stopped with 0.05 M ethylenediaminetetraacetic acid (EDTA), followed by standard ethanol precipitation. The fragmented products were finally resuspended in H_2_O at ~1 μg/μ1, and subjected to immunoprecipitation and m^6^A sequencing.

### RNA immunoprecipitation (IP)

Fragmented RNA (2.5 mg total RNA) was incubated for 2 h at 4 °C with 5 μg of affinity purified anti-m^6^A polyclonal antibody (Synaptic Systems, Göttingen, Germany) in IPP buffer (150 mM NaCl, 0.1% NP-40, 10 mM Tris–HCl, pH 7.4). The mixture was then immunoprecipitated by incubation with protein-A beads (Repligen, Waltham, MA, USA) at 4 °C for an additional 2 h. After extensive washing, bound RNA was eluted from the beads with 0.5 mg/ml *N* ^6^-methyladenosine (Sigma-Aldrich, St Louis, MO, USA) in IPP buffer and ethanol precipitated. The RNA was resuspended in H_2_O and used for library generation with mRNA sequencing kit (Illumina Inc., San Diego, CA, USA).

### Dot blot assays

Dot blot experiments were performed to compare m^6^A enrichment with input RNA before sequencing. 10 ng IP RNA and 10 ng input RNA (without immunoprecipitation) were spotted onto a nylon membrane (Hybond-N+). The membrane was dried and cross-linked twice with 200 000 μJ/cm^2^ UV. The cross-linked membrane was rinsed with PBS + 0.1% Tween-20 for 5 min and blocked in 3% (w/v) BSA in PBS + 0.1% Tween-20 for 1 h. The membrane was finally transferred into blocking solution supplemented with anti-m^6^A antibody (SYnaptic SYstems) diluted 1:1000 and incubated overnight at 4 °C. The antibody exposed membrane was washed thrice with PBS + 0.1% Tween-20 for a total of 30 min and transferred into blocking solution supplemented with HRP-linked secondary antibody diluted 1:2000. This was incubated for 1 h at room temperature, and washed thrice with PBS + 0.1% Tween-20. Peroxidase activity was detected with ECL (Western Lightning Plus-ECL, Perkin-Elmer) or SuperSignal West Femto Chemiluminescent Substrate (Thermo Scientific) and signal detection was done with the ChemiDoc XRS system (BioRad).

### High throughput m^6^A and input RNA sequencing

High throughput m^6^A and input RNA sequencing of two samples from each of muscle and adipose tissues was performed on the Hi Seq3000 sequencing system (Illumina Inc., San Diego, CA, USA), using the Genomic sequencing kit V2 (Illumina Inc., San Diego, CA, USA) as per the manufacturer’s instructions. Approximately 2.5 μg fragmented total RNA was reserved and used for the input RNA-seq before the IP experiments. Thus, m^6^A-seq and input RNA-seq were parallel and their data were mutually comparable in this study. RNA integrity number (RIN) was estimated using a Nanodrop 2000 UV vis (Thermo Fisher Scientific, Wilmington, NC, USA). The quality control (QC) tests were done by Agilent Technologies (Santa Clara, CA, USA). All RNA sequencing of two samples from each of muscle and adipose tissues was performed on the same sequencer at the same batch.

### Data analysis

#### Preprocessing sequencing reads and reads alignment

Sequence data analysis was performed according to the procedure described by Dominissini et al. [10] and Meyer et al. [23]. Briefly, raw data from IP RNA-seq and input RNA-seq were firstly both trimmed by Trim Galore (version 0.3.7) to remove the adapter and low quality data (Q < 25), and the reads shorter than 50 bp were discarded [49]. After this preprocessing, high reads were first aligned against the pig reference genome (Sscrofa10.2, Ensembl *Sus scrofa*) using Tophat software (version 2.0.14) with default parameter [50], and all reads that mapped to multiple genomic regions were discarded. Only reads that uniquely mapped to the reference sequences were analyzed further for m^6^A modification peaks. The read depth distribution was estimated using an *ad hoc R* program.

#### Identification of m^6^A modification peaks

m^6^A modification peaks were called using MACS software (version 1.4.2) with Mfold parameters set to 10, 30 and a *P*-value cutoff of 1e-5, and only one tag, which was optimal for detecting enriched regions was retained [51]; meanwhile, input RNA-seq data were used as background when calling peaks. Peaks that shared at least 50% overlapping lengths were defined as recurrent peaks.

#### Discernment of m^6^A topological patterns

Distribution of m^6^A sites in the different regions of the transcripts was estimated by Dominissini et al.’s method [10]. The consensus m^6^A of motif sequences were identified using Hypergeometric Optimization of Motif EnRichment software [52] with modification: approximately 1,000 the highest m^6^A peaks and approximately 50 nt length around each m^6^A peak were used for deduction of the consensus m^6^A motif sequences. The overall m^6^A distributing patterns were discerned by this method: a gene was splitted in to 60 bins. The read depth of each bin was normalized by per 1 kb per 1 Mb data, then the normalized depth was used to plot the patterns.

#### Analysis of common and differential peaks for tissues or breeds

For a peak to be classified as tissue- or breed- specific, it was assumed not to overlap (<50% overlapping length) any peak of the other tissue or breed; meanwhile we defined a peak which appears on both tissues or breeds as common peak (≥50% overlapping lengths). Dynamic methylated peaks (DMPs), which showing altered intensity in some of the common peaks were judged by MAnorm model [53], and peaks with *P* ≤ 0.01 and normalized change fold ≥ 2 were regarded as DMPs.

#### Multi-layer gene expression and function analysis

Gene expression levels were measured as numbers of input-sequencing reads per kilo bases of exon model in a gene per million uniquely mapped reads (RPKM) and calculated by Cufflinks software (version 2.2.1) [50]. Differentially expressed genes (DEGs) between muscle and adipose tissues were identified with Cuffdiff program (version 2.2.1) [50], and genes with *P* ≤ 0.05 and normalized change fold ≥ 2 were regarded as DEGs. Gene Ontology (GO) analysis was performed using DAVID bioinformatics, based on the GO consortium database and the hypergeometric distribution tes performed by R [54, 55].

#### Statistical analysis and graphics

All statistical analyses (unless stated otherwise) were performed using R and Perl packages for Statistical Computing. Most of the figures were produced using the ggplot2 package (R) [56]. Sequence logos were prepared using SeqLogo [57].

## Additional files

Additional file 1: Figure S1. Dot blot analysis demonstrates antibody specificity for m^6^A. **Figure S2.** The motif sequence for m^6^A-containing peak regions. **Figure S3.** Outline of the common and specific m^6^A peaks among three breeds. **Figure S4.** Gene ontology analysis of the breed specifically methylated genes. (DOCX 922 kb)

Additional file 2: Table S1. Summary of sequence data and read alignment statistics. **Table S2.** m^6^A density of transcripts with RPKM > 2. **Table S3.** Analysis of the correlation between gene expression difference and m^6^A methylation modification. (DOCX 24 kb)

Additional file 3: **data 1.** m^6^A peaks and related genes in muscle and adipose tissues. (XLS 3209 kb)

Additional file 4: **data 2.** Common m^6^A peaks in both muscle and adipose tissues, related genes and GO enrichment. (XLS 1971 kb)

Additional file 5: **data 3.** Nucleus-related genes in PeakStop and GO enrichment. (XLS 1428 kb)

Additional file 6: **data 4.** Different peaks related methylated genes in muscle and adipose tissues. (XLS 2947 kb)

Additional file 7: **data 5.** Go analysis of tissue-differentially methylated genes. (XLS 1292 kb)

Additional file 8: **data 6.** m^6^A peaks and related genes in among three breeds. (XLSX 2851 kb)

Additional file 9: **data 7.** Different peaks related methylated genes among three breeds. (XLS 1836 kb)

Additional file 10: **data 8.** Go analysis of breed-specific and -dynamic genes among three breeds. (XLS 2825 kb)

## Abbreviations

3′-UTRs: 3′-untranslated regions
5′-UTRs: 5′-untranslated regions
BDMGs: Breed dynamic methylated genes
BSMGs: Breed-specific methylated genes
CDS: Coding sequence
CREB: cAMP responsive element-binding protein gene
FTO: Fat mass and obesity-associated protein
GO: Gene ontology
HLCS: Holocarboxylase synthetase
IGSF: Immunoglobulin superfamily
IP: Immunoprecipitation
JAG1: Cardiac transcription factor Jagged1
LA: Landrace adipose tissue
LD: Landrace
LM: Landrace muscle tissue
m^6^A: *N* ^*6*^-methyladenosine
MAP3K14: Mitogen-activated protein kinase 14
MeRIP-Seq: Methylated RNA immunoprecipitation with next-generation sequencing
RC: Rongchang pig
SMGs: Selectively methylated genes
TDMGs: Tissue dynamic methylated genes
TSMGs: Tissue specifically methylated genes
WB: Wild boar
ZNF: Zinc finger protein.
