# Supplementary material for: Transcriptome-wide N6-methyladenosine methylome profiling of porcine muscle and adipose tissues reveals a potential mechanism for transcriptional regulation and differential methylation pattern
Source: BMC Genomics. 2017 Apr 28;18:336. doi: 10.1186/s12864-017-3719-1 (PMC5410061; doi:10.1186/s12864-017-3719-1)
Supplement: Supplementary file 1 — Dot blot analysis demonstrates antibody specificity for m6A. Figure S2. The motif sequence for m6A-containing peak regions. Figure S3. Outline of the common and specific m6A peaks among three breeds. Figure S4. Gene ontology analysis of the breed specifically methylated genes. (DOCX 922 kb) [file 12864_2017_3719_MOESM1_ESM.docx]

Supplementary Figure

Transcriptome-wide *N^6^*-methyladenosine methylome profiling of porcine muscle and adipose tissues reveals a potential mechanism for transcriptional regulation and differential methylation pattern

Xuelian Tao^1,†^, Jianning Chen^1,†^, Yanzhi Jiang^1,†,^*^,^**, Yingying Wei^1^, Yan Chen^1^, Huaming Xu^1^, Li Zhu^2^, Guoqing Tang^2^, Mingzhou Li^2^, Anan Jiang^2^, Surong Shuai^2^, Lin Bai^2^, Haifeng Liu^2^, Jideng Ma^2^, Long Jin^2^, Anxiang Wen^1^, Qin Wang^1^, Guangxiang Zhu^1^, Meng Xie^1^, Jiayun Wu^1^, Tao He^1^, Chunyu Huang^3^, Xiang Gao^3^ and Xuewei Li^2,^**


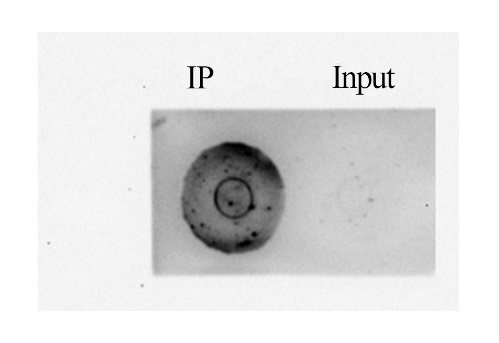
**Supplementary Figure S1.** Dot blot analysis demonstrates antibody specificity for m^6^A. The membranes were spotted with 10 ng of m^6^A RNA and input RNA, respectively.

| **LM** |  |  |  | **LA** |  |  |  |
| --- | --- | --- | --- | --- | --- | --- | --- |
| Motif | *P*-value | % of Targets | % of Background | Motif | *P*-value | % of Targets | % of Background |
| 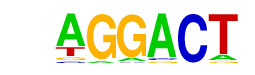 | 1e-65 | 70.00 | 40.00 | 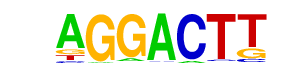 | 1e-59 | 39.60 | 15.07 |
| 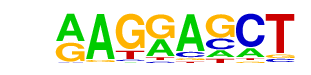 | 1e-28 | 24.50 | 10.33 | 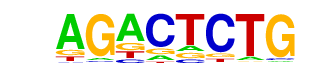 | 1e-56 | 46.00 | 20.43 |
| 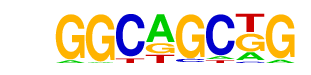 | 1e-24 | 24.90 | 11.44 | 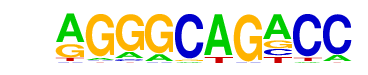 | 1e-23 | 10.50 | 2.56 |
| **Supplementary Figure S2.** The motif sequence for m^6^A-containing peak regions. Analysis was performed by using Hypergeometric Optimization of Motif EnRichment software. LM and LA mean muscle and adipose tissue, respectively. | | | | | | | |
|  | | | | | | | |

**
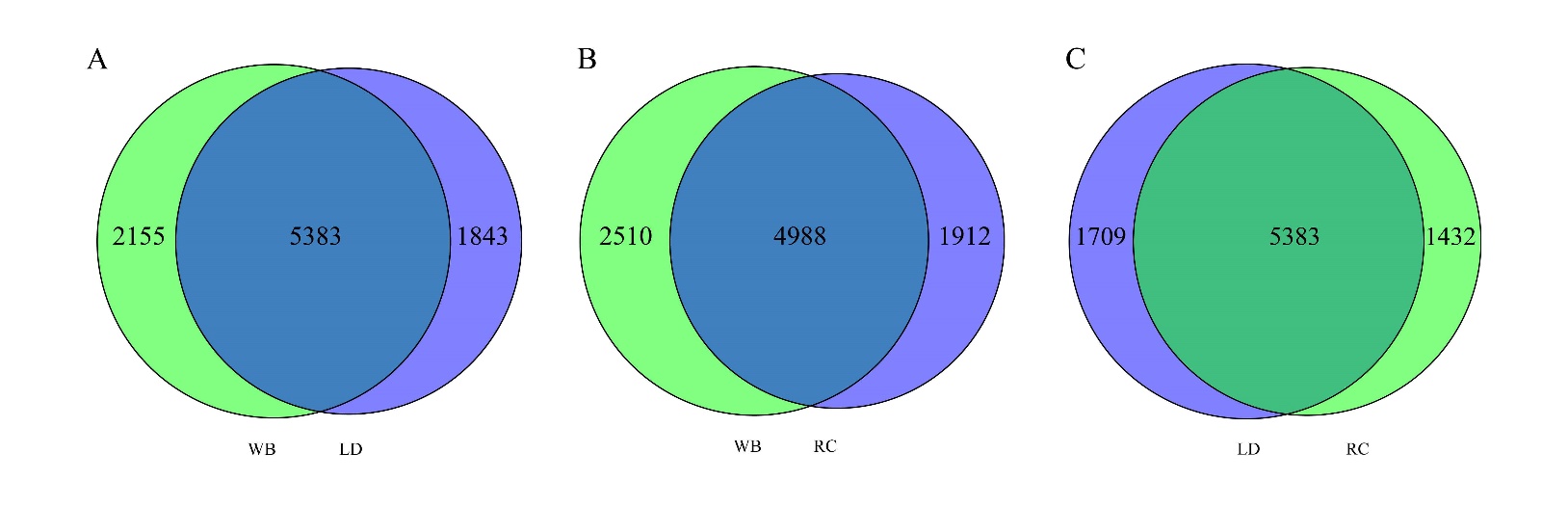
**

**Supplementary Figure S3.** Outline of the common and specific m^6^A peaks among three breeds. **(A)** Number of the common and specific m^6^A peaks between WB and LD. WB means the Wild boar and LD means the Landrace pig, and as follow same. **(B)** Number of the common and specific m^6^A peaks between WB and RC. WB means the Wild boar and RC means the Rongchang pig, and as follow same. **(C)** Number of the common and specific m^6^A peaks between LD and RC.

**
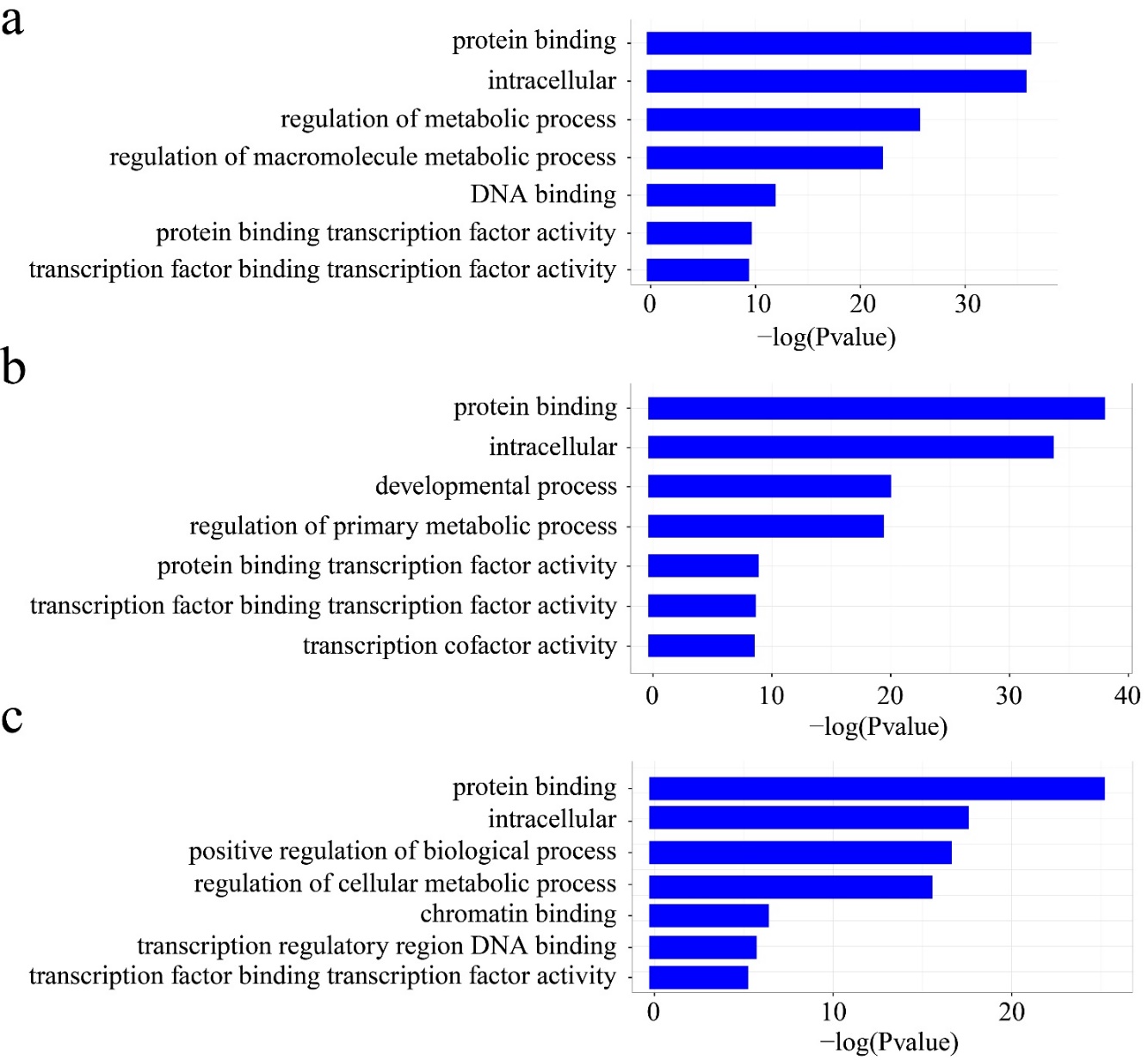
**

**Supplementary Figure S4.** Gene ontology analysis of the breed specifically methylated genes. **(a)** Gene ontology analysis of the breed specifically methylated genes between WB and LD. WB means the Wild boar and LD means the Landrace pig, and as follow same. **(b)** Gene ontology analysis of the breed specifically methylated genes between WB and RC. RC means the Rongchang pigs, and as follow same. **(c)** Gene ontology analysis of the breed specifically methylated genes between RC and LD.
