# Supplementary material for: Transcriptome-wide N6-methyladenosine methylome profiling of porcine muscle and adipose tissues reveals a potential mechanism for transcriptional regulation and differential methylation pattern
Source: BMC Genomics. 2017 Apr 28;18:336. doi: 10.1186/s12864-017-3719-1 (PMC5410061; doi:10.1186/s12864-017-3719-1)
Supplement: Supplementary file 2 — Summary of sequence data and read alignment statistics. Table S2. m6A density of transcripts with RPKM > 2. Table S3. Analysis of the correlation between gene expression difference and m6A methylation modification. (DOCX 24 kb) [file 12864_2017_3719_MOESM2_ESM.docx]

Supplementary Table

Transcriptome-wide *N^6^*-methyladenosine methylome profiling of porcine muscle and adipose tissues reveals a potential mechanism for transcriptional regulation and differential methylation pattern

Xuelian Tao^1,†^, Jianning Chen^1,†^, Yanzhi Jiang^1,†,^*^,^**, Yingying Wei^1^, Yan Chen^1^, Huaming Xu^1^, Li Zhu^2^, Guoqing Tang^2^, Mingzhou Li^2^, Anan Jiang^2^, Surong Shuai^2^, Lin Bai^2^, Haifeng Liu^2^, Jideng Ma^2^, Long Jin^2^, Anxiang Wen^1^, Qin Wang^1^, Guangxiang Zhu^1^, Meng Xie^1^, Jiayun Wu^1^, Tao He^1^, Chunyu Huang^3^, Xiang Gao^3^ and Xuewei Li^2,^**

| **Supplementary Table S1.** Summary of sequence data and read alignment statistics | | | | | | | | |
| --- | --- | --- | --- | --- | --- | --- | --- | --- |
|  | Samples ID | Raw reads | Clean reads | Reads Uniquely mapped to genome | Reads uniquely mapped to junction | Total reads uniquely mapped (%) | m^6^A peaks/m^6^A modified genes | Expressed gene |
| IP-Seq | LM1 | 37668689 | 37491007 | 23349194 | 3507026 | 26856220 (71.63%) | 7131/5864 |  |
|  | LM2 | 38414403 | 37877720 | 24190230 | 3337489 | 27527719 (72.68%) | 6603/5364 |  |
|  | LA1 | 34590648 | 34342607 | 23275535 | 2351071 | 25626606 (74.62%) | 3982/3303 |  |
|  | LA2 | 30970021 | 30673630 | 21087357 | 1843812 | 22931169 (74.76%) | 3575/2974 |  |
| input-Seq | LM1 | 24342227 | 24272185 | 15667450 | 3736404 | 19403854 (79.94%) |  | 15815 |
|  | LM2 | 23905070 | 23812305 | 15502501 | 3756793 | 19249294 (80.84%) |  | 15980 |
|  | LA1 | 21649296 | 21597668 | 14126392 | 2958280 | 17084672 (79.10%) |  | 17015 |
|  | LA2 | 24142684 | 24061985 | 16204343 | 3086867 | 19291210(80.17%) |  | 17258 |
| **Note:** LM1 and LM2 mean the sample 1 and sample 2 of muscle tissue from Landrace pigs, respectively. LA1 and LA2 mean the sample 1 and sample 2 of adipose tissue from Landrace pigs, respectively. | | | | | | | | |

| **Supplementary Table S2.** m^6^A density of transcripts with RPKM > 2 | | | | | | |
| --- | --- | --- | --- | --- | --- | --- |
| Tissue | Peaks | Genes (RPKM>2) | Total length | Average peak density (m^6^A sites/gene) | Average peak density  (m^6^A sites/100knt) | |
| LM | 5872 | 10452 | 445832250 | 0.562 | | 1.32 |
| LA | 2826 | 11106 | 472418230 | 0.254 | | 0.59 |
| Note: LM and LA mean muscle and adipose tissue, respectively. | | | | | | |

| **Supplementary Table S3.** Analysis of the correlation between gene expression difference and m^6^A methylation modification | | | | | | | | | | | | | |
| --- | --- | --- | --- | --- | --- | --- | --- | --- | --- | --- | --- | --- | --- |
| LA *vs.* LM | Total | | | High expression group in LA | | | | | High expression group in LM | | | | |
|  | differential expression gene | Peak ( LA) | peak ( LM) | High expression gene ( LA) | Peak (LA) | Peak ( LM) | Significant factor (LA) | *P* value | High expression gene ( LM) | Peak ( LA) | Peak ( LM) | Significant factor (LM) | *P* value |
|  | 6262 | 807 | 1593 | 3264 | 495 | 581 | 1.6817882 | <0.001 | 2998 | 312 | 1012 | 1.6431745 | <0.001 |
| Note: LM and LA mean muscle and adipose tissue, respectively. | | | | | | | | | | | | | |
